# Identification of a Coding Sequence and Structure Modeling of a Glycine-Rich RNA-Binding Protein (CmGRP1) from *Chelidonium majus* L.

**DOI:** 10.1007/s11105-012-0510-y

**Published:** 2012-09-13

**Authors:** Robert Nawrot, Łukasz Tomaszewski, Anna Czerwoniec, Anna Goździcka-Józefiak

**Affiliations:** 1 Department of Molecular Virology, Institute of Experimental Biology, Faculty of Biology, Adam Mickiewicz University in Poznań, Umultowska 89, 61-614 Poznań, Poland; 2 Bioinformatics Laboratory, Institute of Molecular Biology and Biotechnology, Faculty of Biology, Adam Mickiewicz University in Poznań, Umultowska 89, 61-614 Poznań, Poland

**Keywords:** Glycine-rich protein, RNA-binding, Papaveraceae, *Chelidonium majus*, Coding sequence, 3D model

## Abstract

The family of glycine-rich plant proteins (GRPs) is a large and complex group of proteins that share, as a common feature, the presence of glycine-rich domains arranged in (Gly)n-X repeats that are suggested to be involved in protein–protein interactions, RNA binding, and nucleolar targeting. These proteins are implicated in several independent physiological processes. Some are components of cell walls of many higher plants, while others are involved in molecular responses to environmental stress, and mediated by post-transcriptional regulatory mechanisms. The goals of this study are to identify the coding sequence of a novel glycine-rich RNA-binding protein from *Chelidonium majus* and to propose its structural model. DNA fragments obtained using degenerate PCR primers showed high sequence identities with glycine-rich RNA-binding protein coding sequences from different plant species. A 439-bp nucleotide sequence is identified coding for a novel polypeptide composed of 146 amino acids, designated as CmGRP1 (*C. majus* glycine-rich protein 1), with a calculated MW of 14,931 Da (NCBI GenBank accession no. HM173636). Using NCBI CDD and GeneSilico MetaServer, a single conserved domain, the RNA recognition motif (RRM), was detected in CmGRP1. The C-terminal region of CmGRP1 is a glycine-rich motif (GGGGxxGxGGGxxG), and it is predicted to be disordered. Based on a 1fxl crystal structure, a 3D model of CmGRP1 is proposed. CmGRP1 can be classified as a class IVa plant GRP, implicated to play a role in plant defense.

## Introduction

Glycine-rich plant proteins (GRPs) constitute a newly described group of proteins with diverse localizations and functions (Sachetto-Martins et al. 2000). They were first described as storage proteins, essentially being utilized as sources of amino acids for plants (Mousavi and Hotta 2005). GRPs are associated with dormancy, and are similar to proteins that are stimulated by abiotic stresses. Some are components of cell walls of higher plants and accumulate in vascular tissues as part of defense mechanisms against pathogens and wounding, and also play an important role in post-transcriptional regulation of gene expression (Ringli et al. 2001; Mousavi and Hotta 2005). More than 150 different GRP genes have been identified following transcriptome or whole genome analysis of sugarcane, eucalyptus, rice and *Arabidopsis* (Mangeon et al. 2009, 2010). Their expression is developmentally regulated, and also induced by various chemical, physical and biological factors (Mousavi and Hotta 2005), such as cold, water stress, drought, wounding, and in response to bacterial, viral and fungal infection (Cornels et al. 2000; Park et al. 2000; Ringli et al. 2001; Wang et al. 2011). GRPs are characterized by the presence of a glycine-rich domain arranged in (Gly)n-X repeats, which are highly flexible and may act in protein–protein interactions (Sachetto-Martins et al. 2000). The presence of additional motifs, as well as the nature of glycine repeats, groups them into four major classes (Bocca et al. 2005). GRPs from class I contain a signal peptide followed by a glycine-rich region with GGX repeats. Most of these proteins are cell wall localized. Class II GRPs may or may not have a signal peptide and contain a glycine-rich region followed by a cysteine-rich region at their C-terminus. Class III GRPs contain proteins with lower glycine content, depicting a great diversity of structures. Class IV GRPs comprise RNA-binding domain proteins. The RNA-binding domain has one RNA recognition motif (RRM) at the amino (N) terminal, followed by a carboxy (C) terminal region composed of up to 70% glycine residues, interrupted mostly by arginine or aromatic amino acid residues, and contains arginine–glycine-rich (RGG) motifs. RGG domains participate in protein–protein interaction and nucleolar targeting. These GRPs may also contain a cold-shock domain (CSD) and zinc fingers (CCHC) (Fusaro et al. 2001; Mangeon et al. 2010). Thus, class IV GRPs can be classified into four different sub-classes based on diversity of domain arrangements. Proteins that contain one RRM motif besides the glycine-rich domain belong to subclass IVa; subclass IVb has one RRM and a CCHC zinc-finger; subclass IVc has a CSD and two or more zinc-fingers; and subclass IVd has two RRMs (Mangeon et al. 2010).

RRMs are found in a variety of RNA-binding proteins, including various heterogeneous nuclear ribonucleoproteins (hnRNPs), proteins implicated in the regulation of alternative splicing, and protein components of small nuclear ribonucleoproteins (snRNPs). The motif also appears in a few single-stranded DNA binding proteins (Albà and Pagès 1998).

*Chelidonium majus* L. (Greater Celandine) belongs to the Papaveraceae family and is a rich source of various biologically active substances. All of them occur in the milky sap—a milky-like orange fluid, similar to latex, isolated from the opium poppy (*Papaver somniferum*) (Decker et al. 2000). Recent findings using two-dimensional electrophoresis (2-DE) and tandem mass spectrometry analysis (LC-MS/MS) have demonstrated that the milky sap of this plant contains about 20 defence-related proteins (Nawrot et al. 2007). One of them was identified as a GRP that binds to nucleic acids, similar to AtGRP2B (glycine-rich protein 2B; DNA binding/nucleic acid binding) with MW of 19.4 kDa (Nawrot et al. 2007).

The goals of this study were to identify the coding sequence of a novel glycine-rich RNA-binding protein from *Chelidonium majus* and propose a model for its structure.

## Materials and Methods

### DNA Isolation and PCR Amplification

Genomic DNA was isolated from 14-day-old *C. majus* seedlings using a DNeasy Plant Mini Kit (Qiagen, Hilden, Germany). To obtain the GRP gene sequence, a polymerase chain reaction (PCR) was carried out using degenerate primers GRP1 (5′-TGYTTYGTNGGNGGNCTNG-3′) and GRP4 (5′-CCNCCNCCRTANCC-3′) designed on the basis of highly conserved regions of GRP (Nomata et al. 2004). PCR was performed in a TGradient thermocycler (Biometra, Göttingen, Germany). Initial DNA denaturation was performed at 95 °C for 5 min, then step 2 denaturation at 95 °C for 15 s, followed by primer annealing at 53.4 °C for 30 s and elongation at 72 °C for 120 s. This cycle of denaturation, annealing and elongation was repeated 35 times and followed by final elongation at 72 °C for 15 min.

### Ligation, Transformation and Sequencing

Purified PCR products were ligated into the vector pGEM-T Easy (Promega, Madison, WI). Competent *Escherichia coli* DH5α cells were transformed with ligation products. Plasmids with inserts were extracted with a QIAprep Plasmid Kit (Qiagen) from white transformed colonies and used as templates for PCR amplification using standard M13 primers (Promega). Inserts were sequenced with an automated 3130× Genetic Analyzer (Applied Biosystems, Foster City, CA) at the Faculty of Biology, Adam Mickiewicz University in Poznań, Poland. DNA sequences were analyzed using VectorNTI (Invitrogen, Carlsbad, CA), NCBI VecScreen, BLASTn and BLASTx tools (http://www.ncbi.nlm.nih.gov).

### Protein Structure Prediction and Modeling

Tertiary structure prediction and fold-recognition was carried out via the GeneSilico MetaServer gateway (Kurowski and Bujnicki 2003). The top-scoring fold-recognition alignments to the structures of the selected template were used as a starting point for homology modeling using the “Frankenstein’s Monster” approach (Kosinski et al. 2003, 2005), comprising cycles of model building, evaluation, realignment in poorly scored regions and merging of the best scoring fragments.

For model evaluation, two model quality assessment programs (MQAPs) were used: MetaMQAP (Pawlowski et al. 2008) and PROQ (Wallner and Elofsson 2003). MQAP scores only predict the deviation of a model from the real structure (the real deviation can be calculated only by comparison with the real structures, which of course are not available). Thus, scores reported in this study that indicate, e.g., ‘very good models’, must be interpreted as estimations or predictions that our models are ‘very good’, and not as ultimate validation of the model quality. However, it should be mentioned that both PROQ and MetaMQAP performed quite well in independent benchmarks and can be regarded as robust predictors.

## Results

### The Search for *C. majus* GRP Coding Sequence

In order to obtain the coding sequence of a putative *C. majus* glycine-rich RNA-binding protein, amplification products were obtained after degenerate PCR using a GRP1f/GRP4r primer pair with a gradient of primer annealing temperature. Primers were designed based on a highly conserved RRM domain of known GRP proteins (Nomata et al. 2004). Due to the low quantity, PCR products were recovered from the agarose gel and served as templates for re-PCR using the same set of primers. Specific re-PCR products were obtained in reactions with primers annealing at 53.4 °C. These were ligated into pGEM-T Easy vector (Promega) and cloned in *E. coli* DH5α cells. Plasmids were extracted from white transformed colonies and used as templates for PCR amplification using standard M13 primers (Promega). The presence of inserts was confirmed for plasmids extracted from six separate colonies. The inserts were sequenced and analysed using bioinformatic tools. Contaminating vector sequences were eliminated using NCBI VecScreen and the resulting 300 bp sequence was further analysed using BLASTn and BLASTp tools (http://www.ncbi.nlm.nih.gov). The full-length GRP gene sequence was obtained by a series of PCR reactions with the use of primers complementary to known fragments of the sequence and to regions upstream and downstream based on the homologous sequences from other plant species. High sequence identity to glycine-rich RNA-binding protein coding sequences from different plants (e.g., *Glycine max*, *Arabidopisis thaliana*) was found (about 73 % maximum identity, 154 total score and E-value of 2e-34). The 439 bp nucleotide sequence coding for the novel GRP (named CmGRP1: *Chelidonium majus* glycine-rich protein 1) composed of 146 amino acids has been submitted to the GenBank database under accession number HM173636 (gi|380709245, protein_id AEE42608). The calculated molecular weight of the novel polypeptide was 14,931 Da.

**Fig. 1:**
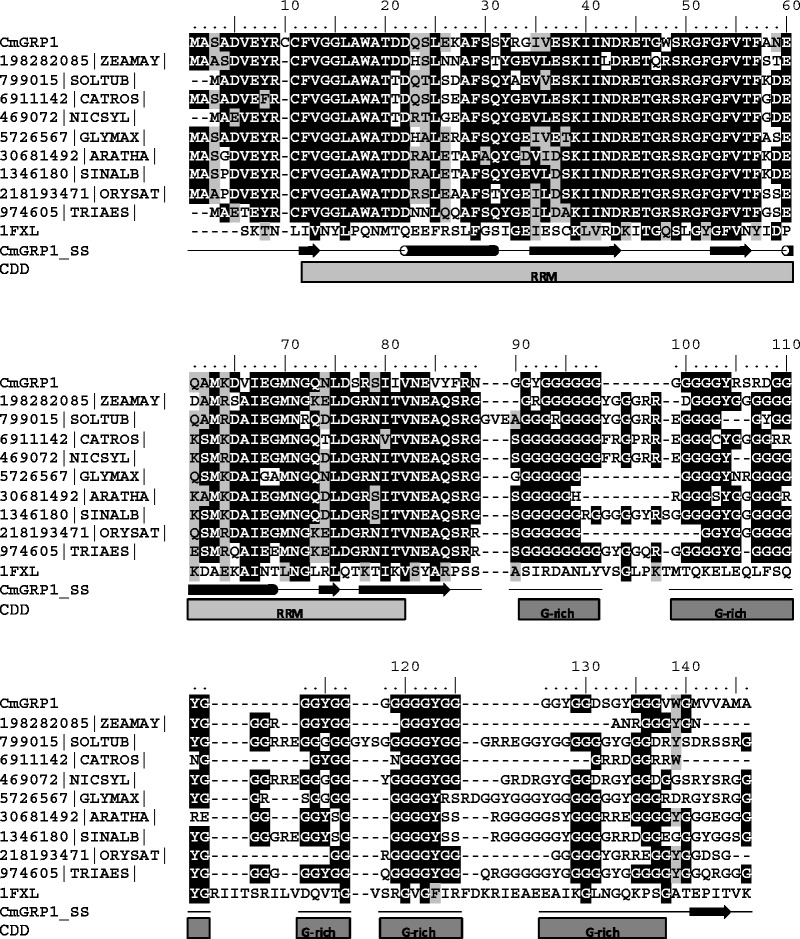
Amino acid sequence alignment of representatives of glycine-rich binding proteins (GRPs). *CmGRP1* Translated amino acid sequence of the *Chelidonium majus* GRP gene obtained in this study. Alignments of amino acid sequences of various known GRPs were created by the ClustalX program. The following sequences were obtained from the GenBank database: glycine-rich RNA binding protein (*Zea mays*); putative glycine rich RNA binding protein (*Solanum tuberosum*); putative glycine-rich RNA binding protein 1 (*Catharanthus roseus*); RNA-binding glycine-rich protein 1-c (*Nicotiana sylvestris*); glycine-rich RNA-binding protein (*Glycine max*); glycine-rich RNA binding protein 7 (*Arabidopsis thaliana*); glycine-rich RNA-binding protein GRP1A (*Sinapis alba*); hypothetical protein OsI_12955 (*Oryza sativa* Indica Group); single-stranded nucleic acid binding protein (*Triticum aestivum*); 1FXL chain A—crystal structure of HUD and AU-rich element of the C-FOS RNA (*Homo sapiens*). NCBI gene identification numbers are presented in the figure with abbreviated genus and species names. Secondary structure prediction for CmGRP1 protein is shown as *arrows* and *tubes* (*strands* and *helices*, respectively). The *blocks* below the sequences indicate conserved motifs (RRM and glycine-rich domain). The positions of conserved residues are colored by two grade shading with *black* (identical residues) and *grey* (similar residues). The *shading* of amino acids in columns is for 60 % threshold values of amino acids similarity

Multiple sequence alignment showed the high sequence similarity of the CmGRP1 translated amino acid sequence to different glycine-rich RNA-binding proteins from different plant species (Fig. 1).

### CmGRP1 Domain Architecture

The presence of conserved domains in the CmGRP1 protein was predicted from information gathered from NCBI CDD (Marchler-Bauer et al. 2009) and GeneSilico MetaServer (Kurowski and Bujnicki 2003; Piszczek et al. 2012). One conserved domain, the RNA recognition motif (RRM, RBD, or RNP domain), was detected. RRM domains are found in a variety of RNA-binding proteins. The RRM domain consists of four strands and two helices arranged in an alpha/beta sandwich, with a third helix present during RNA binding in some cases (SCOP: Fold–Ferredoxin-like). The C-terminal region of the CmGRP1 protein is a glycine-rich motif (GGGGxxGxGGGxxG) and is predicted to be disordered. Based on the classification of plant GRPs, CmGRP1 can be classified into the IVa class (Mangeon et al. 2010).

**Fig. 2:**
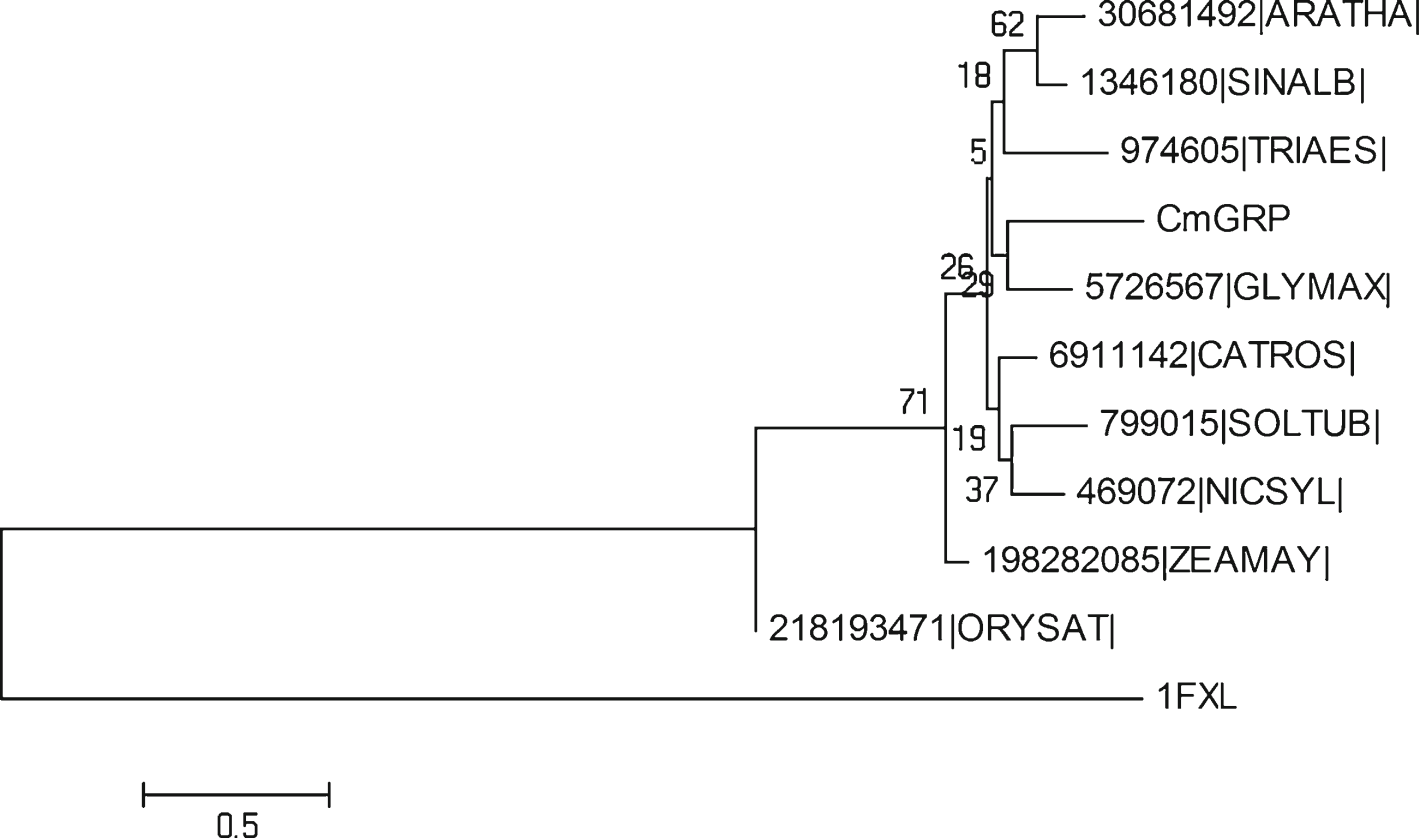
Phylogenetic tree of selected homologs of CmGRP1. Selected sequences are indicated by their abbreviated genus and species names (e.g., ARATHA for *Arabidopsis thaliana*) and the NCBI gene identification (GI) number. See Fig. 1 legend for all genus and species names. Bootstrap support for different nodes is shown

Sequence similarities between selected homologue sequences are shown in Fig. 1. On the basis of this alignment, a preliminary tree was calculated using the neighbor-joining approach implemented in MEGA (Kumar et al. 2004) (Fig. 2). CmGRP1 has a high sequence identity (80 %) and similarity (87 %) to the glycine-rich RNA-binding protein from *Glycine max*, and AtGRP7 from *A. thaliana* (76 % and 87 %, respectively) (Fig. 2).

### CmGRP1 Protein Structure Prediction and Modeling

Based on the fold-recognition (FR) alignment proposed by GeneSilico MetaServer, template structures were selected [PDB: 1 × 5s chain A—solution structure of RRM domain in A18 hnRNP (*Homo sapiens*), mgenthreader-score 3e-05, PDB: 1fxl chain A—crystal structure of HUD and an AU-rich element of the C-FOS RNA (*Homo sapiens*), pdbblast-score 6e-21, blastp-score 3e-08 and 1rk8 chain A—structure of the cytosolic protein PYM bound to the Mago-Y14 core of the exon junction complex (*Drosophila melanogaster*), hhsearch-score 99.94]. The sequence identity in the aligned regions of CmGRP1 and 1fxl, 1x5s and 1rk8 is 36 %, 45 % and 18 %, respectively. All templates are classified as alpha and beta proteins with a ferredoxin-like fold belonging to RNA-binding domain superfamily (SCOP). This allows us to employ a homology modeling approach for constructing 3D protein structures.

**Fig. 3:**
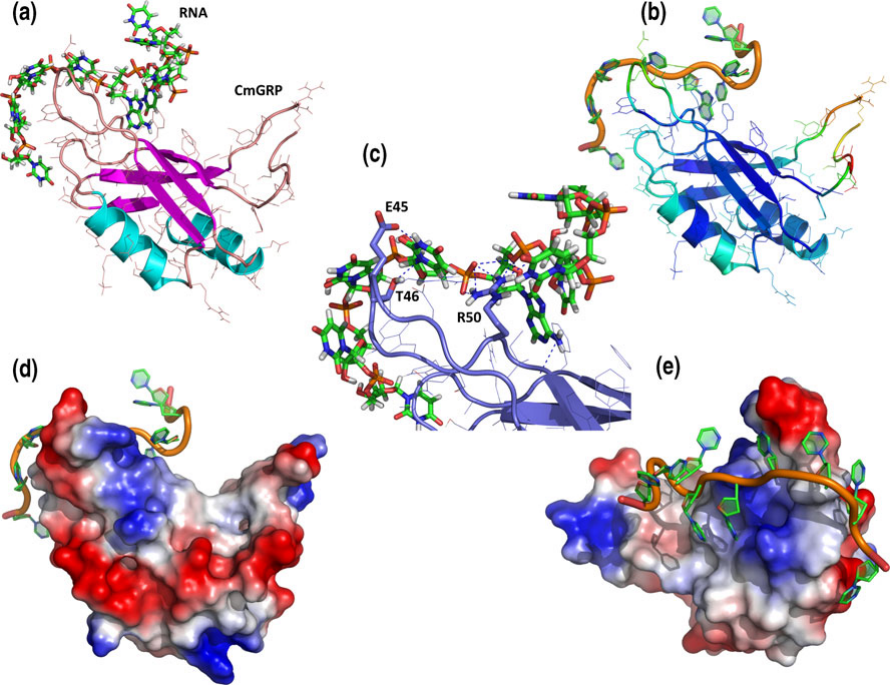
Model of the CmGRP1 protein in a complex with an RNA molecule. All representations of a given protein are shown to the same scale, the RNA molecule is from a 1fxl crystal structure. **a** Model in a ribbon representation with side chains. **b** Model in a ribbon representation, colored according to the predicted local deviation from the real structure (i.e., the predicted error of the model), as calculated by MetaMQAP. *Blue* low predicted deviation of Cα atoms down to 0 Å, *red* unreliable regions with deviation >5 Å, *green*-to-*orange* intermediate values. **c** Fragment or protein and RNA molecule with polar contacts. **d**, **e** Model in two different orientations in the surface representation, colored according to the distribution of the electrostatic surface potential calculated with ABPS (PyMol). *Blue* positively charged regions, *red* negatively charged regions

The modeled structure of the N-terminal fragment (1–89 aa, RRM domain) of protein CmGRP1 was evaluated as a ‘fairly good model’ by the PROQ method for model quality prediction [predicted LGscore: 2.378 (LGscore is −log of a *P*-value)]. The MetaMQAP method predicted that the overall GDT_TS score (global distance test total score—a measure of similarity between two protein structures with identical amino acid sequences, but different tertiary structures) of the model with respect to the native structure is 75.3 and RMSD 1.8, indicating that this model can be used to deduce functional inferences. Figure 3 (panel B) illustrates the predicted quality of different regions of the protein structure.

For CmGRP1, sequence predictions of protein–RNA interaction were conducted. Methods implemented on GeneSilico MetaServer suggested the following amino acids: 46–50, 88–140 (glycine-rich and disordered region). To confirm the predicted and present structures in the template, the Haddock protein-RNA interaction method was used (Haddock web server; de Vries et al. 2010). A RNA molecule from the 1fxl crystal structure was docked flexibly to the CmGRP1 model (Wang and Tanaka Hall 2001). The best model from a highly represented cluster (HADDOCK score: −63.2 +/− 6.4, RMSD from the overall lowest-energy structure: 1.6 +/− 1.0, Van der Waals energy: −32.9 +/− 6.5, electrostatic energy: −128.4 +/− 9.8, desolvation energy: −6.4 +/− 4.2, restraint violation energy: 18.5 +/− 16.61, buried surface area: 997.6 +/− 110.6) was selected (Fig. 3). Detailed interactions for protein region 43–50 as well as polar contacts between side chains (E45, T46, R50) and RNA are shown in Fig. 3c.

## Discussion

Glycine-rich RNA-binding proteins (GRRBPs), which are found primarily in plants as well as in some primate species, are classified on the basis of the presence of a C-terminal glycine enriched region and have extremely diverse and independent functions. Many members of this protein family have a function in stress responses (Kim et al. 2010; Mangeon et al. 2010; Wang et al. 2011). The calculated MW of the novel polypeptide identified here was ca. 15 kDa (14,931 Da). Based on its domain architecture (Fig. 1), CmGRP1 was suggested to belong to class IVa of plant GRPs, which are implicated to play a role in plant defense (Mangeon et al. 2010). CmGRP1 has a high sequence identity (76 %) and similarity (87 %) to AtGRP7 from *A. thaliana* (Figs. 1, 2). GRRBPs AtGRP7 and AtGRP8 were upregulated rapidly in response to peroxide-induced oxidative stress (Schmidt et al. 2009). Another example of the involvement of GRPs in plant defense comes from studies on the interaction between *Arabidopsis thaliana* and *Pseudomonas syringae*. Biochemical analysis indicates that two Class IVa GRPs, AtGRP7 and AtGRP8, are ribosylated in vitro by a type III effector protein during infection by *P. syringae*, resulting in quelled plant immunity. This type III effector is a mono-ADP-ribosyltransferase (HopU1-His) that targets RNA-binding proteins presenting RRM motifs as substrates (Fu et al. 2007).

Using GeneSilico MetaServer, a 3D model of CmGRP1 was proposed on the basis of the 1fxl chain A crystal structure (Fig. 3). HuD, a 167AA protein, binds to adenosine-uridine (AU)-rich elements (AREs) in the 3′ untranslated regions of many short-lived mRNAs, thereby stabilizing them (Wang and Tanaka Hall 2001). Hu proteins contain three highly conserved RRM domains. The first two RRMs are in tandem and are necessary and sufficient for binding to ARE (Wang and Tanaka Hall 2001). For CmGRP1, sequence predictions for protein-RNA interaction were conducted based on the HuD protein in complex with an 11-nucleotide fragment of a class I ARE (the c-fos ARE). An RNA molecule from the 1fxl crystal structure was docked flexibly to the CmGRP1 model confirming the quality of the model (Fig. 3; Wang and Tanaka Hall 2001).

CmGRP1 contains an RRM, found in a variety of canonical RNA-binding proteins. These include hnRNPs implicated in the regulation of alternative splicing, and protein components of snRNPs—central players in mRNA splicing. The motif also appears in a few single-stranded DNA-binding proteins. The RRM structure consists of four strands and two helices arranged in an alpha/beta sandwich, and a third helix in some cases present during RNA-binding (Albà and Pagès 1998). RRM has two highly conserved ribonucleoprotein (RNP) motif sequences. RNP-1 consists of eight amino acid residues and RNP-2 contains six amino acid residues (Nomata et al. 2004). Amino acid residues in RNP-1, RNP-2 and glycine-rich domains are involved in RNA binding (Nomata et al. 2004). RNA-binding proteins are ubiquitous cellular proteins that regulate gene expression mainly at the post-transcriptional level, which involves pre-mRNA splicing, nucleocytoplasmic mRNA transport, mRNA stability and decay, and translation (Kim et al. 2010). It is possible that GRRBPs, as members of RNA-binding proteins (RBPs), act as regulators of RNA processing and/or stability for mRNAs that are highly expressed during stress conditions (Albà and Pagès 1998; Zhang et al. 2011). This, in turn, would lead to the accumulation of stress-related proteins and secondary metabolites with a protective function. RNA is structurally very flexible and proteins, named RNA chaperones, can assist RNAs in reaching their functionally active states in vivo, e.g., by facilitation or prevention of RNA–RNA interactions (Lorković 2009).

In this study, we identified a 439-bp nucleotide sequence coding for a novel polypeptide, named CmGRP1, composed of 146 amino acids from *C. majus*. The protein could be classified into class IVa of plant GRPs, which have a role in plant defense. A 3D model of CmGRP1 based on the 1fxl chain A crystal structure was proposed and showed a good correlation of CmGRP1 with the RNA molecule. These results could form the basis for further studies on the possible correlation of the nucleolytic activity of *C. majus* milky sap with presence of nucleic acid-binding proteins and their role in plant defense.
